# To What Extent Does Arginine Reduce the Risk of Developing Necrotizing Enterocolitis?

**DOI:** 10.7759/cureus.45813

**Published:** 2023-09-23

**Authors:** Minoosh Nasef, Hadhami Ben Turkia, Ali M Haider Ali, Esam Mahdawi, Arun Nair

**Affiliations:** 1 Neonatology, King Hamad University Hospital, Muharraq, BHR; 2 Pediatrics, King Hamad University Hospital, Muharraq, BHR; 3 Obstetrics and Gynaecology, King Hamad University Hospital, Muharraq, BHR; 4 Pediatrics, Saint Peter’s University Hospital, Somerset, USA

**Keywords:** prevention, l-arginine, amino acids, neonate, necrotizing enterocolitis

## Abstract

Necrotizing enterocolitis (NEC) and neonatal sepsis are polar opposite diseases that are commonly encountered in the NICU. Concerning the frequency of these pathologies, NEC is regarded as being a much rarer condition, whereas neonatal sepsis is slightly more commonly encountered. However, neonatal sepsis can present with varying clinical presentations and, if caught late, can be detrimental to the patient. Many different modes of therapies have been studied for both conditions at different levels of pathologies, from a microscopic to a macroscopic level, leading to an assessment of treatment approaches.

With the different ongoing treatment protocols being studied, one such therapy under investigation that does stand out is the use of L-arginine in both conditions. The L-arginine, being an essential amino acid, has many basic biological roles in developing neonates. It mainly involves the production of nitric oxide (NO), a potent vasodilator, which is particularly important in the development of vasculature in almost every organ. In premature infants, poorly developed vasculature makes them more susceptible to injury, therefore increasing the risk of diseases such as NEC and the severity of diseases such as neonatal sepsis.

By assessing the uses of L-arginine and its application towards treating conditions like NEC and neonatal sepsis, we aim to identify its potential benefits as a treatment and its potential applications in clinical practice by understanding its basic functions and role in the pathophysiology of NEC and neonatal sepsis.

## Introduction and background

Necrotizing enterocolitis (NEC) is a gastrointestinal condition associated with increased morbidity and mortality, especially among premature infants. Regarded as an inflammatory disease with several hypothesized theories of pathogenesis, it is frequently associated with sepsis and results in dreadful complications such as peritonitis and intestinal perforation. The classical form of NEC in a premature newborn usually appears around 29 to 32 weeks, commonly presenting symptoms such as abdominal distension, difficulty in feeding, episodes of apnea, and hematochezia [1].

Theories on how NEC develops have been studied from many different perspectives, including a hematological, biochemical, and pathological point of view. From a biochemical standpoint, it is important to note the multitude of functions performed by the intestinal cells. One of the substances secreted by intestinal epithelial cells is arginine, which is an amino acid important for the development and protection of the colon via the synthesis of nitric oxide (NO). Arginine plays a crucial role in situations where the colon is susceptible to ischemia, such as in the case of prematurity [2]. Low plasma arginine levels in preterm newborns have been linked to an increased risk of NEC. Several studies have revealed that supplementation with L-arginine should be considered as an innovative and potentially cost-effective technique for preventing NEC [3].

Decreased levels of L-arginine in preterm newborns have been identified, and this emphasizes the fact that arginine metabolism continues to play a key role in the regulation of gut and systemic immunity. It has been discovered that arginine deficiency increases the susceptibility of preterm neonates to serious infections [4]. Alterations in the metabolism of L-arginine may be linked to metabolic changes seen during sepsis and infection [5].

Neonatal sepsis is still a major source of morbidity and mortality worldwide. Early detection of newborns at high risk allows for prompt treatment and a better overall outcome. Kadir et al. found that the overproduction of NO, which is triggered by infection and inflammation, can be cytotoxic to host cells and is a key component of the complex immuno-inflammatory response. This suggests that NO may be used as an independent marker to assess the severity of neonatal sepsis [6].

During sepsis, protein degradation is increased out of necessity to sustain arginine delivery. In a septic state, endogenous de novo arginine generation from citrulline and exogenous intake of arginine from food are decreased, while arginine catabolism is significantly increased by enhanced use of arginine via the arginase and NO pathways. This results in an overall decreased availability of arginine in the plasma. Subsequently, arginine may be regarded as an essential amino acid in sepsis, and supplementation may enhance microcirculation and protein anabolism [7].

At the time of admission to an intensive care unit, Duke et al. indicate that children with sepsis syndrome and septic shock demonstrated enhanced activity of the L-arginine NO pathway, as determined by combined serum nitrate and nitrite levels, in comparison to a control group of non-septic children [8]. Because of their involvement in cellular proliferation, differentiation, migration, and survival, peptides like glutamine, arginine, and citrulline emerge as possible agents for modulating intestinal inflammation and repair [9].

The goal of this review is to offer an overview of recent literature that analyzes the extent to which L-arginine can be used to treat NEC and newborn sepsis. Our study aims to focus on the physiology and mechanism of action of L-arginine in the body and the role it plays in the pathologies of NEC and neonatal sepsis. Furthermore, we will discuss how L-arginine has been used in clinical practice in the management of neonatal conditions. To conclude, we will highlight the most important metabolic pathways of arginine in neonatal health and how they can be altered to be an addition to the routine management of neonatal conditions such as NEC and sepsis.

## Review

Physiology and pathophysiology of L-arginine

General Functions of L-Arginine

The L-arginine is a semi-essential, basic amino acid used for a number of cellular and biological processes, including the bioavailability of chemical intermediates that replenish the Krebs cycle [10]. Arginine is an essential substrate in humans as an intermediate of the urea cycle, in addition to its anaplerotic role through conversion to glutamate and subsequently alpha-keto-glutarate [11].

Arginine is a well-known carrier in the process of generating nitrogenous waste through the metabolic pathway of the urea cycle. The arginase enzyme is responsible for converting arginine to ornithine and urea in the final step of the pathway. This enables urea to be present for excretion, thereby generating ornithine to re-enter the cycle. Controversy exists regarding the source of endogenous arginine, as some existing literature suggests that a significant amount of arginine comes from its synthesis via the urea cycle, while other studies have demonstrated that there is no net synthesis of arginine by the liver [12].

This vital amino acid is significantly involved in performing numerous biological roles. Arginine has a role in acid-base balance, as the urea cycle is a significant source of bicarbonate consumption [13] and is critical for maintaining acid-base homeostasis [14]. Additionally, arginine is critically important for T-cell proliferation [15] and acts as a substrate for NO production, which is key for the host immune response and defenses [16]. Collagen production is a crucial element of wound healing, and arginine is a vital building block for this process. Another notable function of arginine is its contribution to NO production, synthesized by vascular endothelial cells that regulate vascular tone and cardiovascular function [17]. Nitric oxide is a key vascular signaling molecule that relaxes and expands the vasculature while lowering platelet susceptibility to pro-aggregating chemicals.

Arginine Circulation and Nutrition in Pregnancy and Fetal Development

Maternal nutrition is vital to placental and fetal development and formation during gestation. Through the decisive role arginine plays in nutrition and metabolism, it serves as a precursor for the production of biologically important substances [13,18]. Evidence is emerging demonstrating the crucial role that arginine plays in reproduction, fetal development, wound healing, maintenance of tissue integrity and immune function, as well as treatment of diseases in pregnancy [13-21]. Maternal plasma arginine concentrations were found to be lower in pregnancies complicated by intrauterine growth retardation (IUGR) [22]. Arginine can directly activate p70 S6 kinase and promote the phosphorylation of 4E-BP1 through the mechanistic target of the rapamycin (mTOR) signaling pathway, resulting in the stimulation of protein synthesis [23]. Arginine has been found to be essential for the urea cycle to eliminate ammonia from the liver and blood. Increased quantities of ammonia are dangerous to the growing fetus. Excess ammonia can be produced through the generation of oxidative stress, high intracellular pH, a decrease in ATP production, a reduction in utero-placental blood flow, and nutrient transportation [24].

Nitric oxide and polyamines both use arginine as a substrate (putrescine, spermine, and spermidine). Both NO and polyamines are recognized to be important for fertilization, implantation, embryonic development, and placental angiogenesis [25]. Since NO is an endothelium-derived relaxing factor, it is essential to the regulation of placental-fetal blood flow. Hence, adequate transfer of nutrients from mother to fetus is maintained through the significant role possibly played by NO. Likewise, polyamines regulate numerous cellular functions, from gene expression to protein synthesis, which performs embryonic and fetal proliferation, growth, and differentiation [26]. For angiogenesis, NO and polyamines are key regulators. Impaired angiogenesis has been reported in endothelial nitric oxide synthase (eNOS) knockout mice [27]. Stunted morphological formation and subsequently decreased amounts of arginine and polyamines were found in conceptus tissues during in vivo knockdown of eNOS [28].

On the other hand, maternal plasma asymmetric dimethylarginine (ADMA) levels are reduced in the early stage of gestation but increase as the pregnancy progresses [29]. Higher levels of NO and the accompanying low availability of ADMA in early pregnancy can be beneficial for physiological adaptation and relaxation of the uterus, which will aid in avoiding disrupted intrauterine growth of the developing fetus. On the contrary, NO-induced uterine relaxation in late pregnancy can be antagonized by physiologically increased ADMA levels, which aid in the preparation of uterine muscle fibers for increased contractile activity during delivery. These findings, in summary, suggest that intricate regulation of several components of the arginine metabolic pathway is essential to promoting a viable pregnancy.

Biosynthesis and Metabolism of Arginine

In humans, beneficial intake through diet (around 4 g to 6 g of arginine per day), citrulline through endogenic synthesis (accounting for 10% to 15% of arginine production in its entirety), and protein relinquishment are known sources of free arginine. These elements account for roughly 80% of the circulating arginine [30,31]. Meat, nuts, eggs, and dairy products are considered to be dietary sources of arginine. Conversion of citrulline to arginine during endogenous de novo synthesis of arginine takes place via a two-step enzymatic activity. This process involves the enzymes argininosuccinate lyase (ASL) and argininosuccinate synthase (ASS) in the intestinal-renal center line [31,17]. Citrulline is synthesized from glutamate, glutamine, and ornithine in the mitochondria of enterocytes. Once released into circulation, the kidneys absorb the citrulline and perform the subsequent steps necessary to produce arginine. In the majority of cell types, including myocytes, neurons, adipocytes, macrophages, enterocytes, and endothelial cells, arginine can be converted from citrulline [31].

Five different groups of enzymes are involved in the breakdown of arginine. These include the following: NO synthases (NOSs) pertaining to the production of NO; arginase I involvement as part of the urea cycle; arginase II for the generation of glutamate, proline, and ornithine; arginine decarboxylase (ADC) for agmatine production in organs such as the brain and kidney; and arginine:glycine amidinotransferase (AGAT) for the synthesis of guanidinoacetate, which is the immediate precursor of creatine [31].

Arginine is responsible for giving rise to NO and citrulline, urea, ornithine, proline, glutamate, agmatine, polyamines, guanidinoacetate, and creatine through these catabolic pathways. On a quantitative note, arginine is primarily oxidized through the arginase pathway. A minimal proportion (<2%) of the metabolized arginine is used in the production of NO or the synthesis of polyamine [30]. Additionally, arginine plays a role in the synthesis of proteins. For instance, if complexity is involved in that biochemical pathway, the substrate competition between NOS and arginase will be considered a significant attribute favoring the availability of intracellular arginine [13].

L-Arginine in Inflammatory Conditions Such as NEC

Premature infants are highly vulnerable to life-threatening infections such as late-onset septicemia and NEC. These can be difficult to detect clinically. A recent multicentre survey suggests that up to 21% of very low birth weight (VLBW; birth weight less than 1500 g) infants encounter at least one episode of late-onset (>72 hours of life) blood culture-proven sepsis [32]. Nitric oxide, which is important in NEC pathogenesis, is derived from arginine and hence provides a protective effect. Suppression of NO might increase the area of intestinal damage to a greater extent, and therefore, arginine may be protective in this circumstance [32]. One of the most prevalent gastrointestinal (GI) emergencies in newborns is NEC. Ischemic death of the mucosal intestine is an important characterization of this disorder. This is related to extreme inflammation, invasion of enteric gas-forming organisms, and dismemberment of gas into the intestinal wall and portal venous system [33]. Necrotizing enterocolitis contributes to considerable long-term death in survivors of neonatal invasive care, even though early recognition and intense treatment of this disorder have improved outcomes. This is specifically seen in preterm VLBW infants. As a result, research efforts have focused on developing therapies that will lessen the disorder's risk and severity.

The pathogenesis of NEC remains unclear. For 90% of cases that occur in preterm infants, the available evidence supports a multifactorial mechanism that requires the concurrent presence of an immature intestinal tract and immune system (increased susceptibility), triggers that lead to dysbiosis (disruption of the normal intestinal bacterial flora or microbiome that result in increased growth of potentially pathogenic bacteria), and an overstated inflammatory response by the host with the liberation of cytokines and chemokines. This results in increased susceptibility due to intestinal and immunologic immaturity as well as trigger events or factors that contribute to changes in the microbiome of the intestinal tract (microbial dysbiosis and/or primary infection) and disruption of the intestinal mucosal barrier and microvasculature. This can be brought on by non-human milk feedings and, rarely, components in human milk that may trigger a sensitivity or allergic response (e.g., food protein-induced enterocolitis syndrome or milk protein allergy); medications that cause intestinal mucosal injury or enhance microbial overgrowth (e.g., antibiotics); circulatory instability; anemia; and hyper-inflammatory responses mediated by toll-like receptor-4 innate immunity that triggers events within the intestinal tract.

Preterm infants are increasingly susceptible to the development of NEC because immunologic and intestinal immaturity result in a susceptible host for potential mucosal injury. The risk of NEC is inversely proportional to gestational age (GA), with the risk being greatest in the extremely preterm infant (GA <28 weeks) [33].

Mucosal defense in the gut is mediated by several interrelated components, comprising a physical, biochemical, and immunologic barrier [34-38]. The impairment of the mucosal barrier allows bacteria to gain access to deeper tissues, resulting in inflammation. Increased susceptibility, which predisposes the preterm infant to NEC, is due to the following: (1) an immature mucosal physical barrier with increased permeability and bacterial penetration into the intestinal wall (translocation) compared with term infants [33,36,38]; (2) a not so fully developed intestinal mucin barrier in the very preterm infant. Mucin, which is secreted by the goblet cells, hampers bacterial epithelial binding and enhances bacterial removal [34-37]; (3) increased permeability most likely due to the immaturity of the composition and function of the tight junctions [38]. Tight junctions between epithelial cells maintain the semipermeable property of the intestinal tract; (5) immature immunity and biochemical protection with diminished concentrations of secretory IgA, the major intestinal immunoprotective antibody, mucosal enzymes (e.g., pepsin and proteases), other protective agents (e.g., defensins, lactoferrin), and increased gastric pH, which promotes bacterial overgrowth [36,37]; (6) immature gut motility and function. Preterm infants have dysfunctional gastric emptying and decreased bowel motility. This results in delayed transit time, which increases bacterial proliferation and overgrowth [37].

Potential triggers and risk factors

It is postulated that trigger events and environmental factors initiate intestinal injury in a vulnerable host (a preterm infant), which prompts a hyper-inflammatory response. There is reputable evidence that microbial dysbiosis and non-human milk feeding are risk factors for NEC [38-40]. In addition, less-known evidence suggests that hyperosmolar agents and histamine type 2 (H2) receptor blockers that increase intestinal pH are also environmental risk factors. The evidence is not clear as to whether there is an association between NEC and primary infection, circulatory instability, or anemia and red blood cell (RBC) transfusion. Through evaluating the report from a meta-analysis of 27 eligible studies with 4649 preterm infants (most were double-blinded RCTs, with one study being single-blinded), it was found that probiotics and arginine exhibited better preventive efficacy than placebo, but only probiotics achieved a considerable decrease in overall risk of mortality out of the five food additives studies [41].

The inclusion criteria for the studies were: NEC incidence, all-cause mortality, mortality related to NEC, sepsis, and hospitalization days. Exclusion criteria were studies focusing on feeding rate, studies without additives, or duplicate studies from the same cohort. The L-arginine is resistant to a wide range of antibiotics, which can help prevent intestinal infections. There was no significant difference with additives in regard to the endpoint of NEC-related mortality. Statistical analysis demonstrated the surface under the ranking curve (SUCRA): a higher value meant better performance of the treatment presented. Arginine and lactoferrin exhibited the highest SUCRA values with respect to NEC incidence [41]. Arginine unfortunately had the worst SUCRA ranking under the outcome of mortality, while lactoferrin had the highest. Excess arginine may be harmful because of excess nitric oxide generation, supported by a poorer SUCRA outcome in regard to mortality when compared with a placebo. Staggering factors that need to be considered are gestational age, birth weight, varied methods of using probiotics, umbilical channeling, and the respective stage of NEC. It is possible that premature infants were underrepresented in this overview and that probiotics have the potential to be the most preferable additive, while the use of arginine in preterm infants should be further justified.

## Conclusions

Necrotizing enterocolitis and neonatal sepsis are two distinct conditions that are encountered in the NICU that have overlapping inflammatory features with potential infectious etiologies. There have been substantial developments in understanding the root causes and risk factors for these conditions, as well as establishing effective treatment protocols. The L-arginine is a potential treatment option that can be beneficial for both conditions, but it is imperative that further clinical studies be conducted to obtain enough evidence regarding its efficacy in order to incorporate L-arginine as a treatment option for NEC or neonatal sepsis.
